# Cough up your lungs

**DOI:** 10.1007/s12471-018-1115-3

**Published:** 2018-04-17

**Authors:** L. A. J. Rammeloo, N. A. Blom

**Affiliations:** 1 Department of Paediatric Cardiology, VU University Medical Center, Amsterdam, The Netherlands; 2 Department of Paediatric Cardiology, Leiden University Medical Center, Leiden, The Netherlands

## Question

**Fig. 1:**
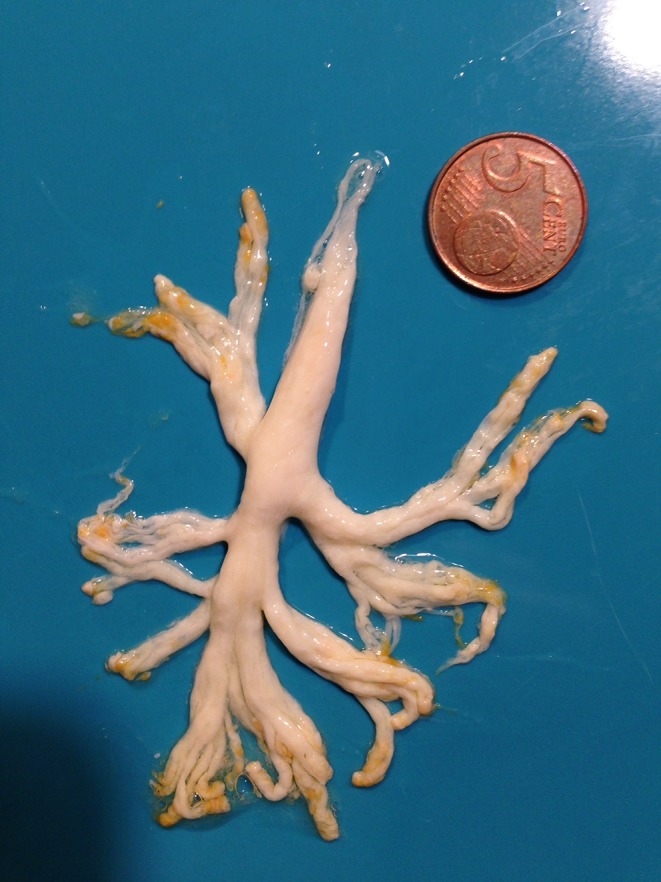
A picture sent by the father showing a expectorated product with a 5 € Cent coin (20 mm) as a size reference

After a frightening coughing episode, a 5-year-old boy with a congenital heart condition expectorated what is shown in the image sent by his father (Fig. 1). He had no fever or signs of chest infection. What is your diagnosis?

## Answer

You will find the answer elsewhere in this issue.

